# Hemophagocytic lymphohistiocytosis in critically ill patients: diagnostic reliability of HLH-2004 criteria and HScore

**DOI:** 10.1186/s13054-020-02941-3

**Published:** 2020-05-24

**Authors:** Cornelia Knaak, Peter Nyvlt, Friederike S. Schuster, Claudia Spies, Patrick Heeren, Thomas Schenk, Felix Balzer, Paul La Rosée, Gritta Janka, Frank M. Brunkhorst, Didier Keh, Gunnar Lachmann

**Affiliations:** 1 Department of Anesthesiology and Operative Intensive Care Medicine (CCM, CVK), Charité – Universitätsmedizin Berlin, corporate member of Freie Universität Berlin, Humboldt-Universität zu Berlin, and Berlin Institute of Health, Berlin, Germany; 2 Department of Hematology and Oncology, Universitätsklinikum Jena, Jena, Germany; 3 Klinik für Innere Medizin II, Schwarzwald-Baar-Klinikum, Villingen-Schwenningen, Germany; 4 Clinic of Pediatric Hematology and Oncology, University Medical Center Eppendorf, Hamburg, Germany; 5 Center for Clinical Studies, Department of Anesthesiology and Intensive Care Medicine, Universitätsklinikum Jena, Jena, Germany; 6 Berlin Institute of Health (BIH), Berlin, Germany

**Keywords:** Hemophagocytic lymphohistiocytosis (HLH), Macrophage activation syndrome (MAS), Hemophagocytic syndrome (HPS), Intensive care unit (ICU), HLH-2004 criteria, HScore, Diagnosis

## Abstract

**Background:**

Hemophagocytic lymphohistiocytosis (HLH) is a rare though often fatal hyperinflammatory syndrome mimicking sepsis in the critically ill. Diagnosis relies on the HLH-2004 criteria and HScore, both of which have been developed in pediatric or adult non-critically ill patients, respectively. Therefore, we aimed to determine the sensitivity and specificity of HLH-2004 criteria and HScore in a cohort of adult critically ill patients.

**Methods:**

In this further analysis of a retrospective observational study, patients ≥ 18 years admitted to at least one adult ICU at Charité – Universitätsmedizin Berlin between January 2006 and August 2018 with hyperferritinemia of ≥ 500 μg/L were included. Patients’ charts were reviewed for clinically diagnosed or suspected HLH. Receiver operating characteristics (ROC) analysis was performed to determine prediction accuracy.

**Results:**

In total, 2623 patients with hyperferritinemia were included, of whom 40 patients had HLH. We found the best prediction accuracy of HLH diagnosis for a cutoff of 4 fulfilled HLH-2004 criteria (95.0% sensitivity and 93.6% specificity) and HScore cutoff of 168 (100% sensitivity and 94.1% specificity). By adjusting HLH-2004 criteria cutoffs of both hyperferritinemia to 3000 μg/L and fever to 38.2 °C, sensitivity and specificity increased to 97.5% and 96.1%, respectively. Both a higher number of fulfilled HLH-2004 criteria [OR 1.513 (95% CI 1.372–1.667); *p* <  0.001] and a higher HScore [OR 1.011 (95% CI 1.009–1.013); *p* <  0.001] were significantly associated with in-hospital mortality.

**Conclusions:**

An HScore cutoff of 168 revealed a sensitivity of 100% and a specificity of 94.1%, thereby providing slightly superior diagnostic accuracy compared to HLH-2004 criteria. Both HLH-2004 criteria and HScore proved to be of good diagnostic accuracy and consequently might be used for HLH diagnosis in critically ill patients.

**Clinical trial registration:**

The study was registered with www.ClinicalTrials.gov (NCT02854943) on August 1, 2016.

## Introduction

Hemophagocytic lymphohistiocytosis (HLH) is a hyperinflammatory syndrome caused by excessive cytokine release, triggered by genetic or acquired overactivation of macrophages, T and natural killer (NK) cells [1]. Clinical presentation may include fever, cytopenias, organomegaly, and hyperferritinemia, none of which are specific for this rare though life-threatening condition [2–4]. As HLH shares similarities with other inflammatory states, e.g., sepsis, its diagnosis is challenged by clinical overlap particularly in the intensive care unit (ICU) [2]. Consequently, HLH is likely to be under-recognized in critically ill patients where evidence for clear definition and correct diagnostic workup is lacking [5]. So far, diagnosis largely relies on data derived from studies conducted in pediatric patients [6]. Henter et al. developed the HLH-2004 criteria whereby a diagnosis of HLH is confirmed if five out of eight criteria are fulfilled [7]. However, these guidelines lack prospective validation in adult HLH patients. Moreover, the specificity of some criteria has been questioned. According to HLH-2004 guidelines, a ferritin ≥ 500 μg/L meets the criterion of hyperferritinemia [7]. However, markedly higher ferritin levels have been seen in adult HLH patients [8]. In fact, we detected best prediction accuracy at a ferritin cutoff level of 9083 μg/L with 92.5% sensitivity and 91.9% specificity for HLH in critically ill patients, thereby providing satisfying discrimination of HLH patients [8].

The HScore published by Fardet et al. [9] calculates a sum score of nine variables allowing to assess the probability of HLH. Each variable was assigned a maximum number varying between 18 and 64 points. The authors found the best discriminatory performance at an HScore of 169 with a sensitivity of 93.0% and specificity of 86.0%. Unlike the HLH-2004 criteria which are composed of parameters derived from a pediatric population, the HScore was developed in an adult cohort including patients aged ≥ 18 years. Yet, only non-ICU patients were included, possibly limiting the tool’s generalizability to critically ill patients. Hence, it is unclear whether HLH-2004 criteria and HScore reliably detect and discriminate HLH in adult critically ill patients. We therefore calculated the sensitivity and specificity of HLH-2004 criteria and HScore, respectively, in a cohort of adult patients admitted to ICUs at an academic medical center.

## Methods

### Patients

This further analysis of a retrospective observational study [8] was conducted at the university hospital Charité – Universitätsmedizin Berlin. Data of patients who were admitted to at least one adult surgical, anesthesiological, or medical ICU between January 2006 and August 2018 were reviewed and extracted from two electronic patient data management systems operated at the Charité – Universitätsmedizin Berlin (COPRA, Sasbachwalden, Germany and SAP, Walldorf, Germany). We included all patients aged ≥ 18 years who had at least one ferritin value measured during ICU stay and hyperferritinemia of at least 500 μg/L according to HLH-2004 criteria [7]. Of all patients included, we extracted data for body temperature, ferritin, blood counts, triglycerides, fibrinogen, soluble interleukin-2 receptor (sIL-2R), and aspartate aminotransferase (AST). Ultrasound, computed tomography (CT) scans and autopsy findings were reviewed to determine the presence of hepatomegaly and/or splenomegaly. Medical reports were screened for evidence of preexisting immunosuppression, while bone marrow findings were reviewed for hemophagocytosis. All variables were recorded at day of maximum ferritin assessment. If no assessment was documented that day, we extended the period to a plausible time range for each parameter according to our protocol (Table 1). Using the obtained data, HLH-2004 criteria and HScore (Supplement Table S1) were determined in all non-HLH patients. To avoid bias by pending parameters at the day of ferritin maximum, we used the highest number of fulfilled HLH-2004 criteria and maximum HScore in all HLH patients. The study period was defined from ICU admission until hospital discharge, transfer, or death.

**Table 1:** Data collection of variables for HLH-2004 criteria and HScore

Variables	Time range with regard to maximum ferritin (when not assessed at day of ferritin maximum)
Hemoglobin, platelets, white blood cell count [min]	± 3 days
Fibrinogen [min]	± 3 days
Triglycerides [max]	± 5 days
Body temperature [max]	± 5 days
AST [max]	± 3 days
NK cell activity, CD107a [max]	± 10 days
sIL-2R	Study period
Hepatomegaly, splenomegaly	Study period
Hemophagocytosis	Study period
Preexisting immunosuppression	Obtained from medical records before study period

*AST* aspartate aminotransferase, *Max* maximum, *Min* minimum, *NK* natural killer cell, *sIL-2R* soluble interleukin-2 receptor

### Diagnosis of HLH

The charts of all included patients were reviewed for clinically diagnosed or suspected HLH. In parallel, we searched for all adult ICU patients diagnosed with ICD-10 codes for HLH (D76.1, D76.2, and D76.3). Only cases with previously suspected or diagnosed HLH by clinicians were reviewed by two HLH experts who confirmed or rejected HLH diagnosis based on HLH-2004 criteria and HScore (Supplement Table S1) while considering patient’s history and clinical presentation, according to current recommendations [6]. Importantly, the diagnosis of HLH was confirmed before HLH-2004 criteria and HScore were determined in all non-HLH patients, i.e., patients who were not previously diagnosed or suspected for HLH by clinicians. Though HLH-2004 criteria and HScore were determined in latter patients, these were not reviewed for HLH by the experts. Of note, HLH patients comprise 7 cases of previously undiagnosed HLH who have been retrospectively detected and described by our research group [5].

### Statistical analysis

Results are reported as median (percentiles) or as counts (relative frequencies) according to their scaling. Comparison of HLH and non-HLH patients was conducted using the non-parametric Mann-Whitney *U* test for continuous variables and the chi-square test for categorical variables. Receiver operating characteristics (ROC) analysis was performed to determine the best prediction accuracy of each diagnostic variable for HLH diagnosis and for fixed combinations. As a post hoc analysis, we rerun ROC analyses for HLH-2004 criteria while raising hyperferritinemia cutoff from 500 to 3000 μg/L based on the lowest ferritin maximum in HLH patients (3102 μg/L). To analyze the best fever cutoff, we reiterated ROC analyses for HLH-2004 criteria using fever cutoffs from 38.0 to 38.5 °C (38.3 °C was used for main analyses as shown in Supplement Table S1). For analysis of hepatomegaly, we extended splenomegaly to spleno- and/or hepatomegaly in another post hoc ROC analyses for HLH-2004 criteria. As a sensitivity analysis, we rerun ROC analyses for HLH-2004 criteria and HScore with restriction to patients with at least 5 assessed HLH-2004 criteria. Multivariable logistic regression analysis was performed to assess associations between HLH-2004 criteria and HScore, respectively, with in-hospital mortality while adjusting for age, sex, body mass index (BMI), and maximum sequential organ failure assessment (SOFA) score. All tests should be understood as constituting exploratory data analysis. No adjustments for multiple testing were made. A two-tailed *P* value < 0.05 was considered statistically significant. All numerical calculations were performed with IBM© SPSS© Statistics, Version 26, © Copyright 1989, 2010 SPSS Inc.

## Results

### Study population and characteristics

**Fig. 1:**
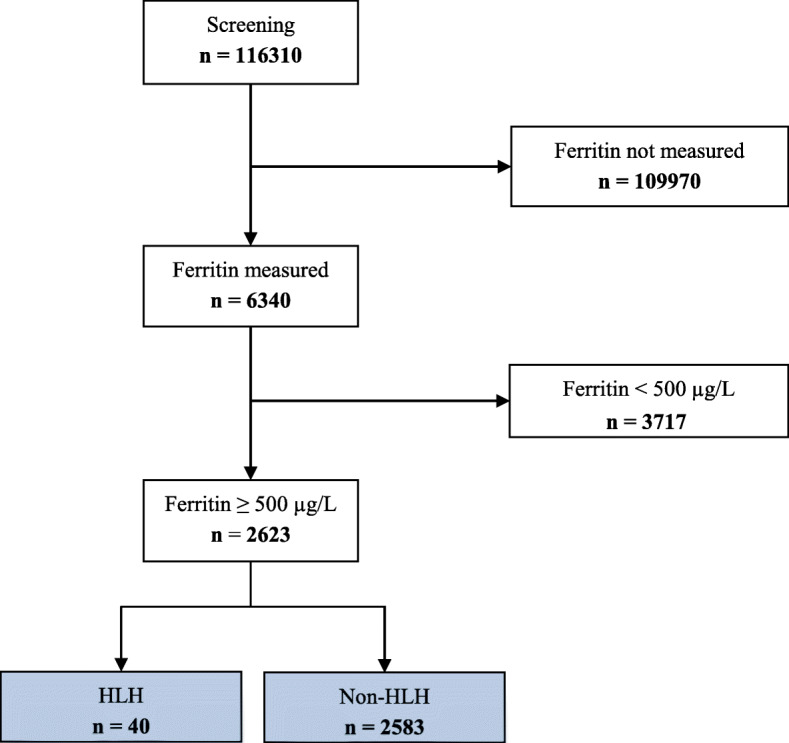
Consort diagram

**Fig. 2:**
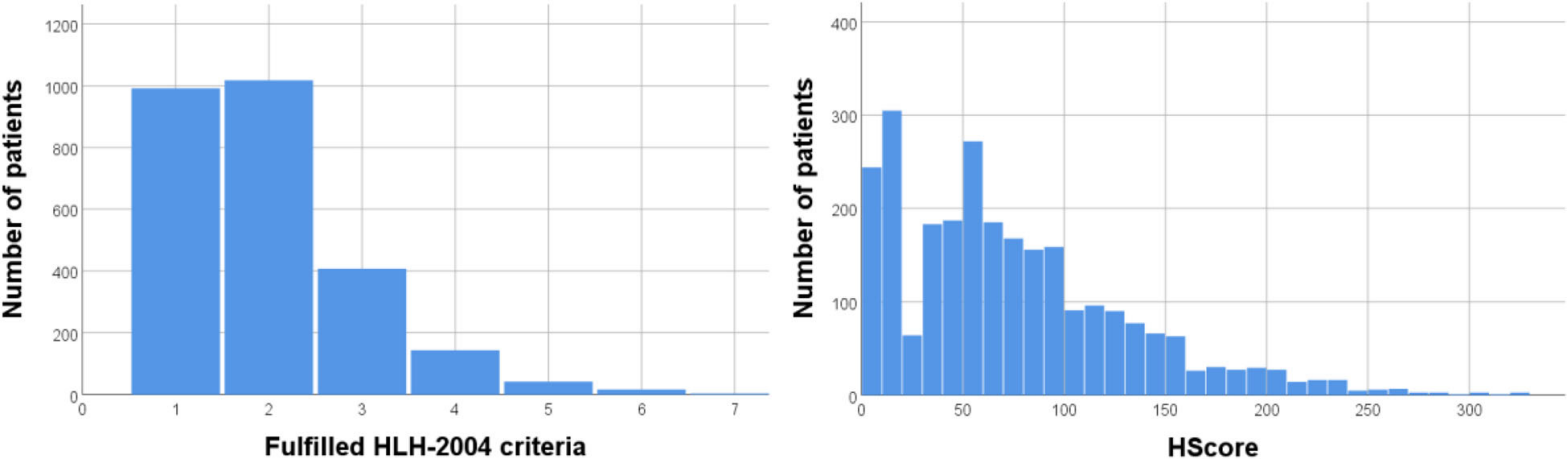
Distribution of fulfilled HLH-2004 criteria and HScore over all patients

Between January 2006 and August 2018, 6340 of 116,310 ICU patients had at least one ferritin measurement during ICU stay and were ≥ 18 years old. Of these, 2623 patients with hyperferritinemia (≥ 500 μg/L) were included into the final analyses. Among those, 50 patients had initially been diagnosed or suspected with HLH by clinicians of whom 40 cases were confirmed by the experts (Fig. 1). The remaining 10 patients (Supplement Table S2) had either low number of fulfilled HLH-2004 criteria (< 4) or low HScore (< 190); in three of the 50 patients, clinical judgment was decisive. Basic patient characteristics, values of HLH-2004 criteria and HScore, and outcome parameters are shown in Table 2. Distribution of fulfilled HLH-2004 criteria and HScore over all patients is shown in Fig. 2. The group of HLH patients has been described in detail previously [4]. The overall cohort of 2623 patients has already been published [8] to analyze hyperferritinemia between patients with HLH, sepsis, septic shock, and other diagnoses.

**Table 2:** Basic patient characteristics, biomarkers, and outcome parameters

Parameters	HLH patients (***n*** = 40)	Non-HLH patients (***n*** = 2583)	***P*** value
Age [years]	47 (33–62)	62 (49–73)	< 0.001^‡^
Male sex [*n*] (%)	26 (65.0%)	1588 (61.5%)	0.650^†^
Body mass index [kg/m^2^]	23.0 (21.0–26.5)	25.0 (22.0–29.0)	0.094^‡^
Sepsis without shock [*n*] (%)	12 (30.0%)	1003 (38.8%)	0.255^†^
Septic shock [*n*] (%)	23 (57.5%)	626 (24.2%)	< 0.001^†^
Hemodialysis [*n*] (%)	29 (72.5%)	1357 (52.5%)	0.012^†^
ECLA/ECMO [*n*] (%)	6 (15.0%)	188 (7.3%)	0.064^†^
ICU admission SOFA score	9 (6–13)	6 (3–9)	< 0.001^‡^
Maximum SOFA score	17 (12–19)	11 (7–15)	< 0.001^‡^
HLH-2004 criteria			
Measured	7 (6–7)	4 (4–5)	< 0.001^‡^
Fulfilled	5 (4–6)	2 (1–2)	< 0.001^‡^
HScore	258 (225–280)	62 (33–101)	< 0.001^‡^
Bi-/pancytopenia [*n*] (%)*, *n* = 40|2582	37 (92.5%)	471 (18.2%)	< 0.001^†^
Hemoglobin [g/dL]	6.9 (6.4–7.6)	8.8 (7.9–9.9)	< 0.001^‡^
Platelet count [/nL]	18 (5–34)	170 (88–270)	< 0.001^‡^
Leukocyte count [/nL]	0.9 (0.2–2.7)	9.0 (6.1–13.3)	< 0.001^‡^
Hypofibrinogenemia or hypertriglyceridemia [*n*] (%), *n* = 40|1517	30 (75.0%)	288 (19.0%)	< 0.001^†^
Fibrinogen [mg/dL], *n* = 39|1144	2.0 (1.0–3.0)	3.8 (2.5–5.3)	< 0.001^‡^
Triglycerides [mg/dL], *n* = 39|855	376 (245–563)	158 (104–247)	< 0.001^‡^
Max. core body temperature [°C], *n* = 40|2452	39.1 (38.5–39.8)	38.2 (37.5–38.9)	< 0.001^†^
Splenomegaly [*n*] (%), *n* = 40|2008	26 (65.0%)	401 (20.0%)	< 0.001^†^
Hepatomegaly [*n*] (%), *n* = 40|2037	23 (57.5%)	328 (16.1%)	< 0.001^†^
Hemophagocytosis [*n*] (%), *n* = 31|221	16 (51.6%)	15 (6.8%)	< 0.001^†^
AST [U/L], *n* = 40|2327	171 (119–498)	47 (26–108)	< 0.001^‡^
Pre-existing immunosuppression [*n*] (%)	30 (75.0%)	728 (28.2%)	< 0.001^†^
ICU duration [d]	20.0 (11.3–37.3)	19.0 (6.0–47.1)	0.522^‡^
In-patient duration [d]	27.7 (18.6–77.4)	38.3 (18.1–76.1)	0.682^‡^
Deceased [*n*]	24 (60.0%)	741 (28.7%)	< 0.001^†^

Diagnostic parameters with *n* representing the number of patients with available data in each group, if not available in all patients; continuous quantities in median with quartiles. CD107a testing as a functional marker for identification of NK cell activity was performed in 4 patients only; however, three showed values within the normal range and one could not be analyzed due to low NK cell count. Values of ferritin and sIL-2R of the cohort were already described in Lachmann et al. [8]

*ECLA* extracorporeal lung assist, *ECMO* extracorporeal membrane oxygenation, *ICU* intensive care unit, *SOFA* sequential organ failure assessment

^‡^*P* values calculated using the Mann-Whitney *U* test

^†^*P* values calculated using the *χ*^2^ test

*Leukopenia was assumed by white blood cell count < 1.67/nL

### Sensitivity and specificity of HLH-2004 criteria and HScore

**Fig. 3:**
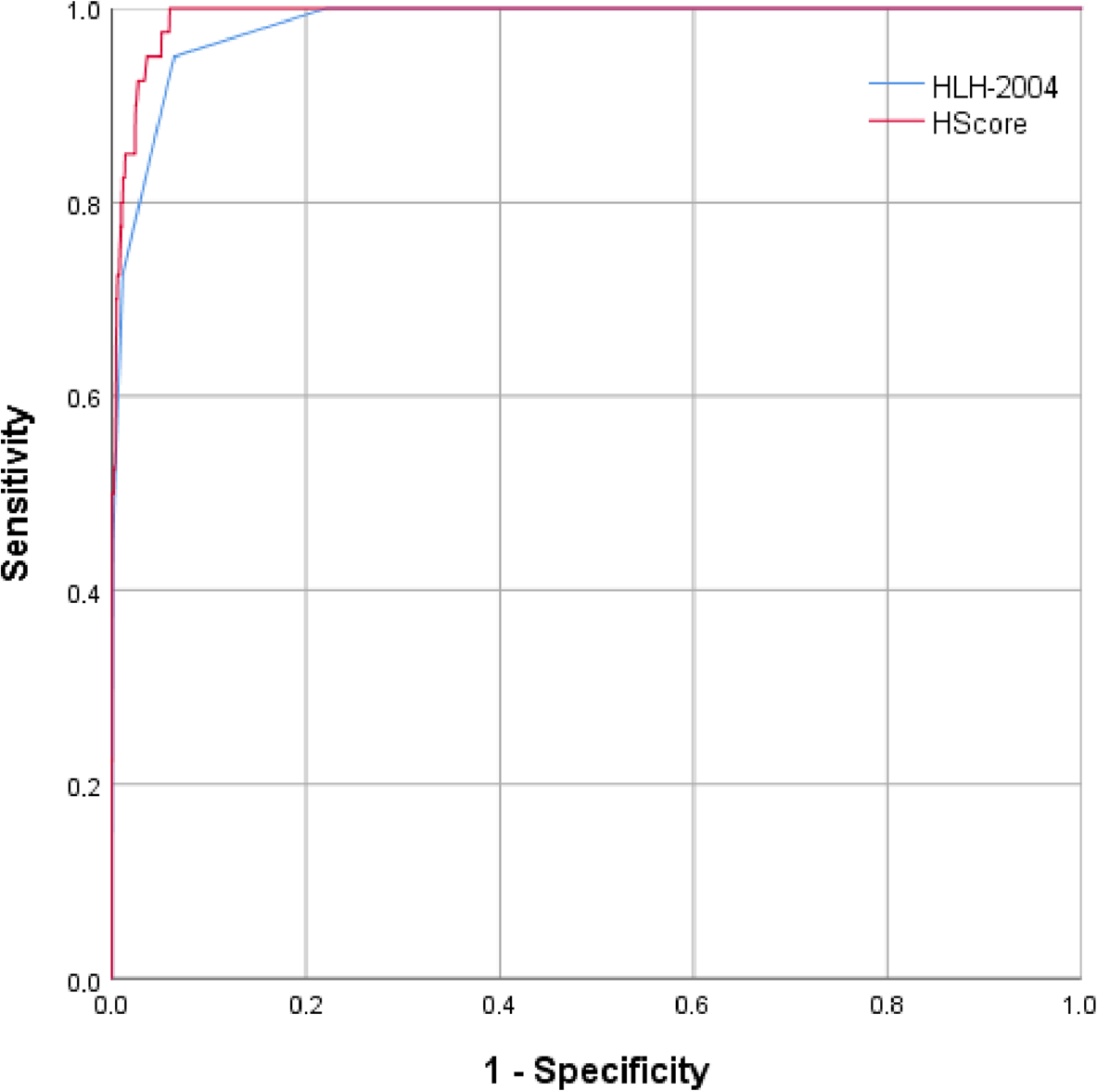
Receiver operating characteristic curves of fulfilled HLH-2004 criteria and HScore

Twenty-nine HLH patients (72.5%) and 33 non-HLH patients (1.3%) fulfilled at least 5 HLH-2004 criteria. ROC curves of fulfilled HLH-2004 criteria and HScore are shown in Fig. 3. The best prediction accuracy for HLH was seen for a cutoff of 4 fulfilled HLH-2004 criteria and an HScore cutoff of 168. The sensitivity and specificity of each criterion are shown in Table 3, while the analyses of ferritin and sIL-2R were already published in Lachmann et al. [8]. When analyses were restricted to patients with at least 5 measured HLH-2004 criteria (*n* = 1303), we found 95.0% sensitivity and 88.0% specificity for the cutoff of 4 fulfilled HLH-2004 criteria (AUC 0.968 (95% CI 0.950–0.986)) as well as 100% sensitivity and 88.6% specificity for the HScore cutoff of 168 (AUC 0.984 (95% CI 0.975–0.993)).

**Table 3:** Sensitivity and specificity of fulfilled HLH-2004 criteria and HScore

Parameters	AUC (95% CI)	Cutoff	Sensitivity/specificity
**HLH-2004 criteria**	0.982 (0.971–0.993)	2	100%/38.4%
		3	100%/77.8%
		4	95.0%/93.6%
		5	72.5%/98.7%
		6	45.0%/99.9%
		7	10.0%/100%
**HScore**	0.992 (0.987–0.996)	140	100%/88.2%
		150	100%/90.7%
		160	100%/93.1%
		168	100%/94.1%
		170	97.5%/94.2%
		180	95.0%/95.3%
		190	95.0%/96.3%
		200	90.0%/97.3%
		210	85.0%/98.3%
Bi-/pancytopenia	0.871 (0.822–0.920)	Yes	92.5%/81.8%
Hypofibrinogenemia or hypertriglyceridemia	0.780 (0.702–0.859)	Yes	75.0%/81.0%
Fibrinogen [g/L]	0.760 (0.677–0.843)	1.5	43.6%/91.1%
		3.1	79.5%/64.5%
Triglycerides [mg/dL]	0.830 (0.776–0.883)	229	84.6%/71.9%
		265	66.7%/77.4%
Max. core body temperature [°C]	0.737 (0.656–0.818)	38.5	80.0%/59.8%
Splenomegaly	0.725 (0.638–0.812)	Yes	65.0%/80.0%
Hemophagocytosis	0.724 (0.611–0.837)	Yes	51.6%/93.2%

Receiver operating characteristics (ROC) analysis to determine the best prediction accuracy of each diagnostic variable for HLH diagnosis. CD107a testing as a functional marker for identification of NK cell activity not shown as performed in 4 patients only. The predictive values of ferritin and sIL-2R of the cohort were already analyzed in Lachmann et al. [8]

*AUC* Area under the curve, *CI* confidence interval

### Post hoc analyses of HLH-2004 criteria cutoffs

When hyperferritinemia cutoff was raised from 500 to 3000 μg/L, the specificity of 4 fulfilled HLH-2004 criteria increased to 96.1%, while sensitivity remained 95.0% (AUC 0.989 (95% CI 0.983–0.996)). By analyses of different fever cutoffs, best prediction accuracy was found for 38.2 °C (97.5% sensitivity and 93.5% specificity for 4 fulfilled HLH-2004 criteria (AUC 0.984 (95% CI 0.975–0.994))). By adjusting cutoffs of both hyperferritinemia to 3000 μg/L and fever to 38.2 °C, the sensitivity and specificity of 4 fulfilled HLH-2004 criteria were 97.5% and 96.1%, respectively (AUC 0.991 (95% CI 0.985–0.996)). Extension of splenomegaly to spleno- and/or hepatomegaly reduced specificity of 4 fulfilled HLH-2004 criteria from 93.6 to 92.2% while the sensitivity of 95.0% was unchanged (AUC 0.981 (95% CI 0.968–0.993)). Analyses of fixed combinations of fulfilled HLH-2004 criteria showed less prediction accuracy compared to independent combinations of at least 4 fulfilled HLH-2004 criteria (Supplement Table S3).

### HLH-2004 criteria and HScore for prediction of mortality

Multivariable logistic regression analysis including age, sex, BMI, and maximum SOFA score as confounders revealed statistically significant associations between in-hospital mortality and fulfilled HLH-2004 criteria or HScore, respectively (Table 4). In-hospital mortality of fulfilled HLH-2004 criteria and HScore strata is shown in Supplement Table S4.

**Table 4:** Multivariable logistic regression analyses for in-hospital mortality

Covariates	HLH-2004 criteria			HScore		
	OR	95% CI	***P*** value	OR	95% CI	***P*** value
Age, years	1.025	1.018–1.031	< 0.001	1.031	1.024–1.038	< 0.001
Sex (male)	0.943	0.778–1.143	0.550	0.897	0.737–1.091	0.276
BMI, kg/m^2^	0.979	0.965–0.994	0.005	0.980	0.965–0.995	0.009
SOFA score max	1.163	1.140–1.185	< 0.001	1.148	1.126–1.171	< 0.001
Fulfilled HLH-2004 criteria	1.513	1.372–1.667	< 0.001	–		
HScore	–			1.011	1.009–1.013	< 0.001

Multivariable logistic regression analyses were performed with in-hospital death as dependent variable

*BMI* body mass index, *CI* confidence interval, *OR* odds ratio, *SOFA* sequential organ failure assessment

## Discussion

This is the largest study investigating the diagnostic performance of HLH-2004 criteria and HScore in an adult intensive care population. We found the best prediction accuracy of HLH diagnosis for a cutoff of 4 fulfilled HLH-2004 criteria (95.0% sensitivity and 93.6% specificity) and an HScore cutoff of 168 (100% sensitivity and 94.1% specificity). Analyses of each single HLH-2004 criterion revealed good sensitivity and specificity for a ferritin cutoff of 9083 μg/L described previously [8] while all other HLH-2004 criteria had unsatisfying predictive ability in our study, including sIL-2R, which was also described previously [8]. The combination of 4 fulfilled HLH-2004 criteria provided better diagnostic accuracy compared to each single criterion. By adjusting HLH-2004 criteria cutoffs of both hyperferritinemia to 3000 μg/L and fever to 38.2 °C, sensitivity and specificity increased to 97.5 and 96.1%, respectively. Our approach to analyze fixed combinations of fulfilled HLH-2004 criteria showed less prediction accuracy compared to independent combinations of at least 4 fulfilled HLH-2004 criteria. Both HLH-2004 criteria and HScore were independently associated with in-hospital mortality.

The HLH-2004 criteria, currently the standard in HLH diagnosis, have been developed in pediatric populations but so far have not been validated in adult patients. According to current recommendations for HLH in adults, HLH diagnosis requires ≥ 5 fulfilled HLH-2004 criteria which should be considered along with patient’s history and clinical presentation [6]. In daily practice, clinical presentation might be suggestive of HLH, while less than 5 out of 8 HLH-2004 criteria are present. Moreover, the diagnostic value of some criteria, e.g., fever, is limited, particularly in critically ill patients where the use of antipyretic agents and devices such as extracorporeal membrane oxygenation (ECMO) and hemodialysis are frequently seen rendering body temperature an unreliable or even invalid parameter. Of note, the cutoff of 4 fulfilled HLH-2004 criteria had the best sensitivity and specificity possibly allowing faster HLH diagnosis, prompt treatment, and thus improved survival. Yet, these findings need further confirmation in prospective studies to validate safe HLH diagnosis in adults with only 4 fulfilled HLH-2004 criteria. Ongoing studies could contribute to improve safe HLH diagnosis in adult critically ill patients [10].

Our analysis of sensitivity and specificity of single HLH-2004 criteria is broadly in line with data reported in pediatric HLH patients. Hypofibrinogenemia is known to have high specificity but rather low sensitivity as only 53% of children with HLH had fibrinogen levels < 1.5 g/L [11]. Also, we found sensitivity for fibrinogen of 1.5 g/L to be at 43.6% while specificity was at 91.1%. In contrast to studies in pediatric populations, sIL-2R proves to be of insufficient diagnostic value in adult patients [8] whereas levels ≥ 2400 U/L in children provided good sensitivity and excellent specificity of 93.0% and 100%, respectively [11]. The diagnostic value of ferritin in the present cohort has been described previously by our research group and appeared as a good screening marker [8]. Importantly, the presence of 4 fulfilled HLH-2004 criteria provides higher sensitivity and specificity for HLH diagnosis than ferritin alone. Of note, sensitivity and specificity of 4 fulfilled HLH-2004 criteria increased to 97.5% and 96.1%, respectively, when cutoffs of both hyperferritinemia and fever were adjusted to 3000 μg/L and 38.2 °C, respectively. In this context it is noteworthy that 5 out of 8 HLH-2004 criteria can be fulfilled in critically ill non-HLH patients. NK cell activity was assessed in four patients only in whom HLH was likely considered as a differential diagnosis. For practical guidance, we recommend assessment of body temperature, cytopenias, ferritin, triglycerides, fibrinogen, splenomegaly, and wherever available sIL-2R. Hemophagocytosis, even though the eponymous feature of HLH with high specificity, is an unreliable diagnostic marker with only poor sensitivity, again particularly in critically ill patients with sepsis [12]. However, the latest recommendations for HLH in adult patients advise bone marrow investigation as it helps to detect occult hemato-oncological malignancies and to differentiate between cytopenias caused by chemotherapy from patients who have actually underlying HLH [6]. The HScore developed by Fardet et al. [9] provides a tool to predict the probability of HLH diagnosis in adults. The authors found the best cutoff at an HScore of 169 yielding 93.0% sensitivity and 86.0% specificity in a cohort of non-ICU patients. Our present study included ICU patients only and revealed an HScore of 168 to have the best sensitivity and specificity of 100% and 94.1%, respectively, thereby providing slightly superior prediction accuracy compared to the HLH-2004 criteria. Importantly, the similar found cutoff underlines the value of the HScore for HLH diagnosis and its reliability in critically ill patients.

One previous study by Meena et al. [13] also analyzed the diagnostic performance of HLH-2004 criteria and HScore in critically ill patients. The authors included 445 patients with ferritin assessment among whom ten were diagnosed with HLH. They reported an HScore of 143.5 for best possible classification and found 5 out of 6 criteria to be the cutoff for HLH-2004 criteria with 70% and sensitivity and 97.2% specificity. However, we present a larger cohort of 2623 patients including 40 HLH cases. Yet, the work by Meena et al. and our study are currently the only data available investigating the diagnostic standard for HLH diagnosis in the adult ICU population.

Both HLH-2004 criteria and HScore were associated with in-hospital mortality suggesting that both indicate disease severity. This relationship has been reported previously by Gualdoni et al. who found increased 30-day mortality correlating with HLH-2004- or HScore-positive patients [14].

Our study has several limitations. As this is a retrospective study, data availability had to rely on patients who had a ferritin assessment during their ICU stay. This might constitute an important selection bias as patients with ferritin assessment might have been more severely ill. For instance, suspicion of inflammation or diagnostic of anemia was likely when ferritin assessment was considered. Thus, our findings might not be generalizable to ICU patients without hyperferritinemia. In addition, not all variables of HLH-2004 criteria and HScore were available in all patients which reflects clinical practice where rather rare diagnostic tests such as NK cell activity might be unavailable. Our study bears a considerable risk that HLH cases could have remained undiagnosed depending on physicians’ expertises, particularly in patients with ≥ 5 fulfilled HLH-2004 criteria.

## Conclusions

This is currently the largest study investigating the diagnostic performance of HLH-2004 criteria and HScore in an adult ICU cohort. Four fulfilled HLH-2004 criteria as cutoff for a diagnosis of HLH had a sensitivity of 95.0% and a specificity of 93.6%. By adjusting cutoffs of both hyperferritinemia to 3000 μg/L and fever to 38.2 °C, sensitivity and specificity increased to 97.5% and 96.1%, respectively. An HScore cutoff of 168 revealed a sensitivity of 100% and a specificity of 94.1%, thereby providing slightly superior diagnostic accuracy compared to HLH-2004 criteria. With regard to single criteria, ferritin demonstrated the best diagnostic performance of all 8 HLH-2004 criteria warranting its use as a reliable screening parameter for HLH diagnosis. Both HLH-2004 criteria and HScore proved to be of good diagnostic accuracy and consequently might be used for HLH diagnosis in critically ill patients.

## Supplementary information

**Additional file 1: Supplemental Table S1.** HLH-2004 criteria and HScore (7, 9). AST*, aspartate aminotransferase;* hb, *hemoglobin;* mM, *mmoles/liter;* plt, *platelets;* U, *Units.***Supplemental Table S2.** Fulfilled HLH-2004 criteria and HScore of suspected HLH patients where HLH was not confirmed. **Supplemental Table S3.** Sensitivity and specificity of fixed combinations of fulfilled HLH-2004 criteria. *Patients with complete obtained data in each category. AUC, Area under the curve; CI, confidence interval. Receiver operating characteristics (ROC) analysis to determine best prediction accuracy of each category (dichotomous variable) for HLH diagnosis.**Supplemental Table S4.** In-hospital mortality of fulfilled HLH-2004 criteria and HScore strata.

## Data Availability

Due to legal restrictions imposed by the data protection commissioner of the Charité – Universitätsmedizin Berlin, public sharing of study data with other researchers or entities is restricted to anonymized data. Requests may be sent to dai-researchdata@charite.de.

## Abbreviations

AST: Aspartate aminotransferase
AUC: Area under the curve
BMI: Body mass index
CI: Confidence interval
CT: Computed tomography
ECLA: Extracorporeal lung assist
ECMO: Extracorporeal membrane oxygenation
Hb: Hemoglobin
HLH: Hemophagocytic lymphohistiocytosis
HPS: Hemophagocytic syndrome
ICD: International classification of diseases
ICU: Intensive care unit
MAS: Macrophage activation syndrome
NK: Natural killer cell
OR: Odds ratio
Plt: Platelets
ROC: Receiver operating characteristics
sIL-2R: Soluble interleukin-2 receptor
SOFA: Sequential organ failure assessment
U: Units
