# Supplementary material for: Hemophagocytic lymphohistiocytosis in critically ill patients: diagnostic reliability of HLH-2004 criteria and HScore
Source: Crit Care. 2020 May 24;24:244. doi: 10.1186/s13054-020-02941-3 (PMC7245825; doi:10.1186/s13054-020-02941-3)
Supplement: Supplementary file 1 — Additional file 1: Supplemental Table S1. HLH-2004 criteria and HScore (7, 9). AST, aspartate aminotransferase; hb, hemoglobin; mM, mmoles/liter; plt, platelets; U, Units.Supplemental Table S2. Fulfilled HLH-2004 criteria and HScore of suspected HLH patients where HLH was not confirmed. Supplemental Table S3. Sensitivity and specificity of fixed combinations of fulfilled HLH-2004 criteria. *Patients with complete obtained data in each category. AUC, Area under the curve; CI, confidence interval. Receiver operating characteristics (ROC) analysis to determine best prediction accuracy of each category (dichotomous variable) for HLH diagnosis.Supplemental Table S4. In-hospital mortality of fulfilled HLH-2004 criteria and HScore strata. [file 13054_2020_2941_MOESM1_ESM.docx]

**Supplement**

**Supplemental Table S1. HLH-2004 criteria and HScore (7, 9).**

| **HLH-2004 criteria: ≥ 5 must be fulfilled** | **HScore: calculated by scoring criteria of each parameter (**[**http://saintantoine.aphp.fr/score/**](http://saintantoine.aphp.fr/score/)**) Best prediction accuracy for HScore 169 (sensitivity 93 %, specificity 86 %)** |
| --- | --- |
| Fever (≥ 38.3 °C)  Splenomegaly  Cytopenias (affecting ≥ 2 of 3 lineages in the peripheral blood):  Hemoglobin <90 g/L  Platelets <100_x 10^9^/L  Neutrophils <1.0_x 10^9^/L  Hypertriglyceridemia and/or hypofibrinogenemia:  Fasting triglycerides ≥ 3.0 mmol/L (i.e., ≥ 265 mg/dl)  Fibrinogen ≤ 1.5 g/L  Hemophagocytosis in bone marrow or spleen or lymph nodes  Low or absent NK-cell activity (according to local laboratory reference)  Ferritin ≥ 500 µg/L  Soluble CD25 (i.e., soluble IL-2 receptor) ≥ 2400 U/ml | Known underlying immunosuppression (no \| yes)  Temperature (< 38.4°C \| 38.4-39.4°C \| > 39.4°C)  Organomegaly (no \| hepato- or splenomegaly \| hepatosplenomegaly)  Cytopenias (1 \| 2 \| 3, hb ≤ 9.2 g/dL, leukocytes ≤ 5,000 /µL, plt ≤ 110000 /µL)  Ferritin (< 2000 µg/L \| 2000-6000 µg/L \| L/>6000 µg/L)  Triglycerides (< 1.5 mM \| 1.5-4 mM \| > 4 mM)  Fibrinogen (> 2.5 g/L \| ≤ 2.5 g/L)  AST (< 30 U/L \| ≥ 30 U/L)  Hemophagocytosis in bone marrow aspirate (no \| yes) |

AST*, aspartate aminotransferase;* hb, *hemoglobin;* mM, *mmoles/liter;* plt, *platelets;* U, *Units.*

**Supplemental Table S2. Fulfilled HLH-2004 criteria and HScore of suspected HLH patients where HLH was not confirmed.**

| **Patient** | **Fulfilled HLH-2004 criteria** | **HScore** |
| --- | --- | --- |
| 1 | 5 | 117 |
| 2 | 2 | 151 |
| 3 | 4 | 141 |
| 4 | 3 | 64 |
| 5 | 2 | 52 |
| 6 | 1 | 61 |
| 7 | 4 | 169 |
| 8 | 3 | 146 |
| 9 | 3 | 182 |
| 10 | 3 | 130 |

**Supplemental Table S3. Sensitivity and specificity of fixed combinations of fulfilled HLH-2004 criteria.**

| **Combination** | **N*** | **Fulfilled** | **AUC  (95 % CI)** | **Sensitivity / Specificity** |
| --- | --- | --- | --- | --- |
| **Bi-/Pancytopenia + Ferritin ≥ 500 µg/L + Fever + Splenomegaly** | 1955 | 89 | 0.732 (0.633 – 0.831) | 50.0 % / 96.4 % |
| **Bi-/Pancytopenia + Ferritin ≥ 500 µg/L + Splenomegaly** | 2048 | 150 | 0.769 (0.676 – 0.862) | 60.0 % / 93.7 % |
| **Bi-/Pancytopenia + Ferritin ≥ 500 µg/L + Fever** | 2492 | 274 | 0.838 (0.762 – 0.914) | 77.5 % / 90.1 % |
| **Bi-/Pancytopenia + Ferritin ≥ 3000 µg/L + Fever + Splenomegaly** | 1955 | 58 | 0.740 (0.641 – 0.840) | 50.0 % / 98.0 % |
| **Bi-/Pancytopenia + Ferritin ≥ 3000 µg/L + Splenomegaly** | 2048 | 83 | 0.785 (0.691 – 0.879) | 60.0 % / 97.1 % |
| **Bi-/Pancytopenia + Ferritin ≥ 3000 µg/L + Fever** | 2492 | 143 | 0.865 (0.788 – 0.941) | 77.5 % / 95.4 % |

**Patients with complete obtained data in each category. AUC, Area under the curve; CI, confidence interval. Receiver operating characteristic (ROC) analysis to determine best prediction accuracy of each category (dichotomous variable) for HLH diagnosis.*

**Supplemental Table S4. In-hospital mortality of fulfilled HLH-2004 criteria and HScore strata.**

| **Parameters** | **Strata** | **N** | **Mortality** |
| --- | --- | --- | --- |
| **HLH-2004 criteria** | 1  2 3 4 5 6 7 | 992  1018 409 142 41 17 4 | 195 (19.7 %)  285 (28.0%) 165(40.3 %) 76 (53.5 %) 28 (68.3 %) 12 (70.6 %) 4 (100 %) |
| **HScore** | 0-29  30-59  60-89  90-119  120-149  150-179  180-209  210-239  240-269  270-299  300-329 | 613  642  509  346  233  120  82  46  18  7  7 | 85 (13.9 %)  128 (19.9 %)  163 (32.0 %)  122 (35.3 %)  108 (46.4 %)  55 (45.8 %)  52 (63.4 %)  32 (69.6 %)  11 (61.1 %)  4 (57.1 %)  5 (71.4 %) |
